# Magnetic Functional Materials: Synthesis, Characterization and Application: A New Open Special Issue in Materials

**DOI:** 10.3390/ma15092999

**Published:** 2022-04-20

**Authors:** Haiou Wang, Yan Wang, Dexin Yang

**Affiliations:** College of Materials and Environmental Engineering, Hangzhou Dianzi University, Hangzhou 310018, China; 202040219@hdu.edu.cn (Y.W.); dy263@hdu.edu.cn (D.Y.)

*Magnetic Functional Materials: Synthesis, Characterization and Application* is a new open Special Issue of *Materials*, which aims to publish original and review papers on new scientific and applied research, and make great contributions to the finding and understanding of magnetic functional materials and related synthesis, fundamentals, characterization, and applications.

The early magnetic materials were mainly silicon steel and ferrite. Since the 1960s, a series of high-performance magnetic functional materials such as amorphous soft magnets, nanocrystalline soft magnets and rare-earth permanent magnets have appeared one after another [1]. Driven by contemporary advanced science and technology, new properties and new phenomena of magnetic functional materials are emerging, and their fields are becoming wider and wider.

In the contemporary information society, energy, information, and materials are the important foundation of production, life, and high technology. Magnetic functional materials are widely used in energy, information, and materials science and technology. There are many kinds of magnetic functional materials, and their progress is rapid. Magnetic functional materials have attracted a great deal of attention regarding their applications. Magnetic behaviors are widespread in a variety of materials, such as metals, ceramics, organics, and emerging 2D materials. Applications of magnetic materials include memories, sensors, magnetic refrigeration, drug delivery, electrochemistry, environmental protection, energy storage, and more.

The research interest of the section *Magnetic Functional Materials: Synthesis, Characterization and Application* includes, but is not limited to, the following: permanent magnets; magnetic functional materials; magnetism in correlated electron systems; memories and sensors devices; magnetic refrigeration; environmental protection; and devices based on magnetic materials.
